# Conditional, genetic disruption of ciliary neurotrophic factor receptors reveals a role in adult motor neuron survival

**DOI:** 10.1111/j.1460-9568.2008.06298.x

**Published:** 2008-06

**Authors:** Nancy Lee, Rachel Robitz, Rebekah J Zurbrugg, Adam M Karpman, Ashley M Mahler, Samantha A Cronier, Rachel Vesey, Rachel P Spearry, Sergei Zolotukhin, A John MacLennan

**Affiliations:** 1 Department of Molecular and Cellular Physiology, University of CincinnatiCincinnati, OH 45267-0576, USA; 2 Division of Cellular and Molecular Therapy, Department of Pediatrics, University of FloridaGainesville, FL 32606-0266, USA

**Keywords:** adeno-associated virus, CNTF receptor α, Cre recombinase, mouse

## Abstract

Indirect evidence suggests that endogenous ciliary neurotrophic factor (CNTF) receptor signaling can promote motor neuron (MN) survival in the adult. If so, proper targeting of this signaling may selectively counteract the effects of adult MN diseases. However, direct evidence for CNTF receptor involvement in adult MN survival is lacking, presumably because the unconditional blockade of the mouse CNTF receptor *in vivo* [through genetic disruption of the essential CNTF receptor α (CNTFRα) gene] leads to uniform perinatal death of the mice. To overcome this limitation, we have developed a method to selectively disrupt CNTF receptor function in a targeted subset of adult MNs that are not required for survival. A ‘floxed CNTFRα’ mouse line was generated and characterized. In addition, an adeno-associated virus (AAV) vector that drives Cre recombinase (Cre) expression was constructed and shown, with reporter mouse lines, to selectively excise floxed genes in facial MNs following its stereotaxic injection into the facial motor nucleus. Adult floxed CNTFRα mice were then injected with the AAV-Cre vector to excise the CNTFRα gene in the targeted MNs. The resulting data indicate that adult CNTF receptor signaling, likely by the MNs themselves, can play an essential role in MN survival. The data further indicate that this role is independent of any developmental contributions CNTF receptor signaling makes to MN survival or function.

## Introduction

Indirect data suggest that endogenous growth factor mechanisms have evolved to promote neuronal survival (e.g. White & Krause, 1993; Cuevas & Gimenez-Gallego, 1997; Kordower *et al.*, 2000; Ozawa *et al.*, 2000). Like endogenous pain control systems, these mechanisms are not completely effective, given that insults such as trauma and disease can still have devastating effects. However, selective enhancement of these mechanisms, to harness their evolved power and specificity, may lead to valuable therapies. Development of such targeted interventions will require a better understanding of how these mechanisms naturally function *in vivo*.

Ciliary neurotrophic factor (CNTF) is one of the most potent neuroprotective factors for developmental motor neurons (MNs) *in vitro* (Lindsay *et al.*, 1994). *In vivo,* exogenously administered CNTF protects MNs following early postnatal axotomy (Sendtner *et al.*, 1990), in genetic models of MN disease (Sendtner *et al.*, 1992; Ikeda *et al.*, 1995; Sagot *et al.*, 1995; Pun *et al.*, 2006), and during developmental naturally occurring MN death (Oppenheim *et al.*, 1991). These pharmacological data raise the promising possibilities that endogenous CNTF receptor signaling may promote MN survival and that manipulating this signaling may be a valuable tool in the treatment of adult MN diseases.

However, amyotrophic lateral sclerosis (ALS) trials with systemic CNTF injections were stopped due to unacceptable side effects (Miller *et al.*, 1996), indicating that any manipulation of CNTF signaling in MN diseases will need to be more specifically targeted. Unfortunately, it is not known where exogenous CNTF acts to protect MNs *in vivo*. Evidence suggests that the effect need not directly involve MN CNTF receptors. For instance, skeletal muscle CNTF receptors maintain muscle (Helgren *et al.*, 1994), thereby raising the possibility that the CNTF indirectly promotes MN survival by maintaining muscle-derived MN survival factor(s). The CNTF may also promote MN survival by increasing muscle-derived, soluble CNTF/CNTF receptor α (CNTFRα) complexes that could enhance MN CNTF receptor signaling (Davis *et al.*, 1993a). In addition, CNTF, at the concentrations employed, can activate leukemia inhibitory factor (LIF) receptors (Saggio *et al.*, 1995), which have been implicated in MN survival (Li *et al.*, 1995).

The CNTF receptor consists of CNTFRα, LIF receptor β (LIFRβ) and gp130, with CNTFRα being unique to CNTF receptors and required for all known forms of CNTF receptor signaling (Davis *et al.*, 1993b; Elson *et al.*, 2000; Derouet *et al.*, 2004). CNTFRα knockout mice uniformly die within 24 h of birth, with a 30–50% reduction in MNs (DeChiara *et al.*, 1995), indicating that endogenous CNTF receptor signaling is essential for embryonic MN survival/development. However, it is not known whether: (i) this reflects an embryonic function of CNTF receptors that is not required in the adult; and (ii) this results from loss of CNTF receptor signaling in MNs or other cell types. These are critical issues for the development of CNTF receptor-related therapeutics because most MN disease symptoms initiate in adulthood and the next generation of treatments will need to be designed to reduce side effects by targeting specific cell types. The present study employed adult-onset CNTFRα gene disruption in MNs to directly address these questions.

## Materials and methods

### Mouse lines

Exons 3–5 of the CNTFRα gene (‘exon1’ containing start codon) were flanked by *lox* P sites (‘floxed’) using previously described methods (Wattler *et al.*, 1999), through contract with Lexicon Genetics. Mice, maintained on a 129/SvEvBrd background, were produced by heterozygote × heterozygote crosses and genotyped by Southern blot or polymerase chain reaction analysis of tail biopsy DNA. ROSA26 and Z/EG mice from Jackson Laboratories and Corrinne Lobe (Sunnybrook and Women's College, Toronto, Canada), respectively, were backcrossed onto the 129SvEvBrd background. Animal procedures were approved by the University of Cincinnati IACUC in accordance with NIH guidelines.

### Adeno-associated virus (AAV)-Cre recombinase (Cre)

Adeno-associated virus (AAV)-Cre was generated with previously described procedures (Zolotukhin *et al.*, 1999). Mice were anesthetized with ketamine/xylazine i.p. and received aseptic, bilateral stereotaxic facial nuclei injections (coordinates from Bregma: AP –5.5, ML ± 1.1, DV −5.7) with a 33-guage Hamilton needle. Each side received 1.5 μL of AAV-Cre [1 × 10^8^ infectious particles/μL in phosphate-buffered saline (PBS) = ‘AAV-Cre’; 1 × 10^7^ infectious particles/μL in PBS = ‘1/10 AAV-Cre’] or PBS at 0.25 μL/min (with 1 min between each 0.25 μL injected and a 10-min wait before needle removal).

### Anatomical procedures

Mice were overdosed with Avertin and transcardially perfused with cold saline followed by 4% para formaldehyde (PFA). Brains were post-fixed overnight in 4% PFA followed by cyroprotection in 30% sucrose with 2.5 mM sodium azide. Coronal, cryostat sections (30 μm) from throughout the facial nucleus were stained with either Cresyl violet (CV), standard Xgal histology or previously described immunohistochemistry (MacLennan *et al.*, 1996). Antibodies recognizing CNTFRα (‘3×’; MacLennan *et al.*, 1996), green fluorescent protein (GFP; Millipore, Temecula, CA, USA) or Cre (Covance, Denver, PA, USA) were visualized through either ABC amplification (Vector Laboratories, Burlingame, CA, USA) and cyanine-3 tyramide (Perkin-Elmer, Waltham, MA, USA), or AlexaFluor-conjugated secondaries (Invitrogen, Carlsbad, CA, USA).

### MN counting

Cresyl violet-stained facial MNs were counted in every fourth section. To correct for cells potentially split in the *z*-dimension, all neurons in focus at the top border of the sections were excluded (optical dissector; Hyman *et al.*, 1998; Hatton & Von Bartheld, 1999). Counts were multiplied by four to estimate total MNs (‘fractionator’). This procedure was validated by counting all facial MNs in two mice and obtaining values, in each case, that were within 3% of the fractionator estimate. As with all processing and analysis, MN counting was conducted by individuals unaware of experimental conditions, including genotype. Statistical analysis consisted of Student's *t*-tests (when only two groups were involved) or anova followed by Bonferroni *post hoc* tests.

## Results

### Characterization of floxed CNTFRα mice

**Fig. 4:**
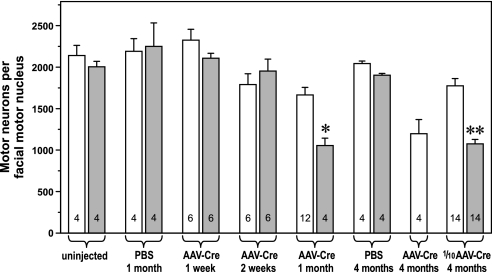
Adeno-associated virus (AAV)-Cre recombinase (Cre) injection into the facial motor nucleus of floxed CNTFRα mice leads to genotype-dependent loss of facial MNs. The mean (±SEM) number of facial MNs in wild-type (open bars) and floxed CNTFRα (solid bars) mice following the indicated treatments. *N* per condition presented in bars. Less than identically treated wild-type controls *P < 0.05; **P < 0.001; less than vehicle (phosphate-buffered saline; PBS)-injected wild-type controls ^‡^*P* < 0.01.

Floxed CNTFRα mice were generated in which Cre-induced removal of the targeted sequence functionally inactivates the CNTFRα gene, based on both the unconditional CNTFRα knockout (DeChiara *et al.*, 1995) and structure–function relationships for related cytokine receptors (Bazan, 1990). Matings of heterozygote floxed mice yielded 24.7% homozygous floxed mice (i.e. 53 of 215; not significantly different from predicted Mendelian frequency). Homozygous floxed mice (referred to simply as floxed mice here) survive with no apparent abnormalities or decrease in CNTFRα expression relative to wild-type mice. Moreover, while embryonic MN survival/development is highly dependent on CNTFRα function, as demonstrated by the loss of these cells in unconditional CNTFRα knockout mice (DeChiara *et al.*, 1995), the floxed mice display no loss of MNs, even at several months of age (see below; Fig. 4). Therefore, as expected, the insertion of the *lox* P sites into introns of the CNTFRα gene has no noticeable effect on CNTFRα function.

**Fig. 1:**
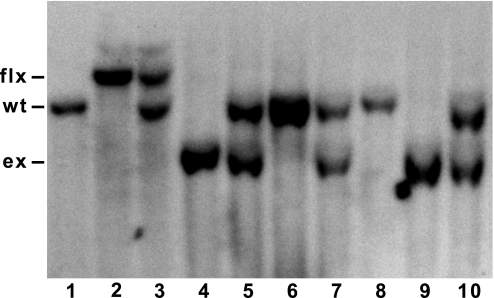
Cre expression leads to CNTFRα gene disruption in floxed CNTFRα mice. Breeding floxed CNTFRα mice with protamine-1-Cre ‘deleter’ mice leads to the designed excision of floxed CNTFRα sequence as indicated by Southern blot bands of predicted size (DNA from tail biopsies). Wild-type (lane 1), homozygous floxed (lane 2), heterozygous floxed (lane 3) and progeny of a heterozygous excised x heterozygous excised cross (lanes 4–10). ex, excised; flx, floxed; wt, wild-type.

Crossing the floxed mice with a ‘deleter’ line that produces floxed gene excision in all cells (protamine 1-Cre mice) resulted in: (i) Southern bands corresponding to the designed Cre-dependent CNTFRα gene excision (Fig. 1); (ii) the expected perinatal death of all homozygous floxed mice, but not heterozygous or wild-type littermates; and (iii) the expected loss (approximately 30%; Lee *et al.*, preliminary data) of facial MNs in homozygous floxed mice, all as seen with universal CNTFRα gene disruption (DeChiara *et al.*, 1995).

### A recombinant AAV vector excising floxed gene sequence in adult MNs

**Fig. 2:**
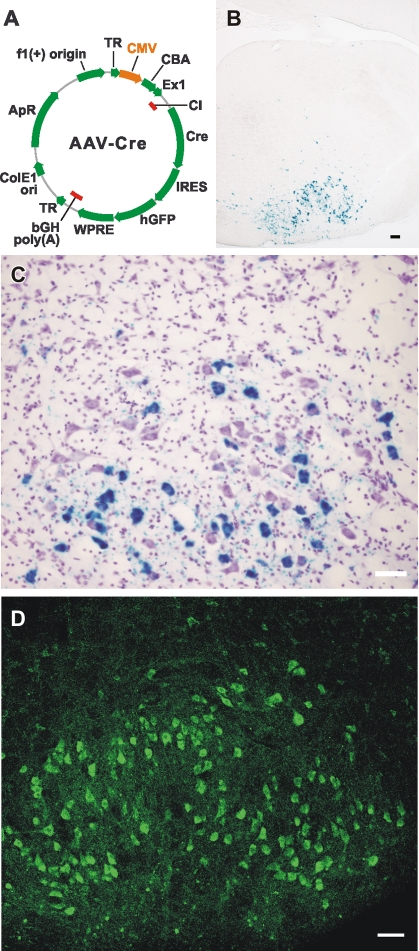
Facial motor nucleus AAV-Cre injection leads to excision of floxed gene sequence in facial MNs. (A) AAV-Cre vector containing a Cre-IRES element-GFP cassette driven by a CMV/chicken β-actin (CBA) promoter and incorporating the woodchuck post-transcriptional regulatory element (WPRE) followed by the rabbit β-globin polyadenylation signal. The whole cassette is flanked by terminal repeat sequences (TR) of AAV-2. (B and C) Adult ROSA26^+/−^ reporter mice injected with AAV-Cre 2 weeks prior to perfusion and Xgal histology. The blue reaction product indicating floxed gene excision is primarily confined to facial MNs [e.g. the cluster of large cells in the bottom center of (B) and in the CV counterstained section in (C)]. Higher magnification indicates that the many small blue specks result from labeling of MN processes (supplementary Figs S1–S4). In contrast, gene excision in facial nucleus astrocytes, through a GFAP-Cre gene construct, does not produce the many small blue specs, but clearly labels CV-stained small cells (presumptive astrocytes) that are not seen with AAV-Cre injection of the nucleus (supplementary Fig. S5). (D) GFP immunohistochemistry of an adult Z/EG^+/−^ reporter mouse perfused 2 weeks after AAV-Cre injection. The reporter signal is once again primarily confined to facial MNs (oval cluster of large cells). It is important to note that any GFP expression from the weak IRES element of AAV-Cre was undetectable in facial MNs following AAV-Cre injection of Z/EG^−/−^ (wild-type) mice, even with GFP immunohistochemical enhancement. Scale bars: 100 μm (B); 50 μm (C); 75 μm (D).

An AAV-2 vector driving Cre expression was constructed (AAV-Cre; Fig. 2A). AAV-2 vectors efficiently transduce neurons leading to long-term, vector-directed expression while eliciting little if any immune response, presumably due to the lack of any viral genes (Muzyczka, 1992). Because AAV-Cre is unable to replicate, Cre is expressed specifically in cells transduced by the injected virus particles.

**Fig. 3:**
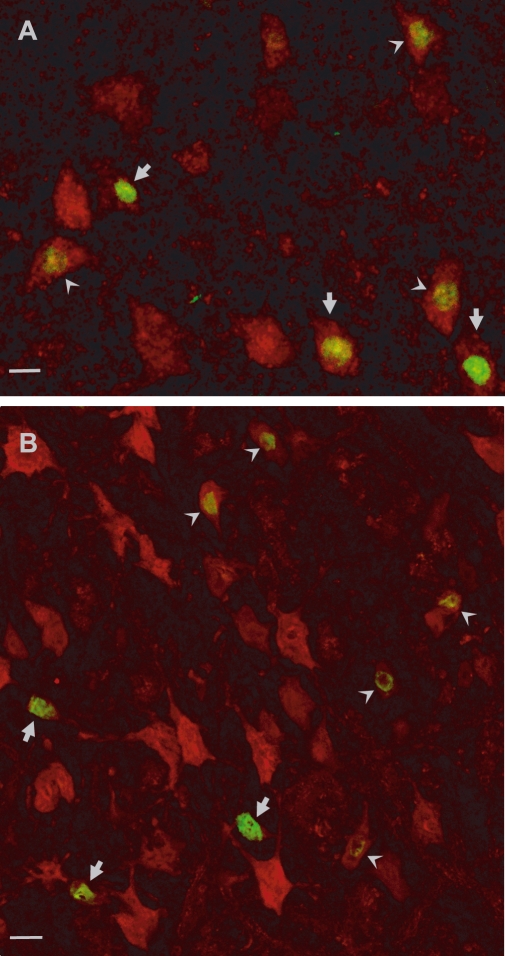
Facial motor nucleus AAV-Cre injection leads to MN-selective expression of Cre. Facial MNs immunohistochemically labeled for expression of either the MN marker, choline acetyltransferase (red in A) or the neuronal marker, NeuN (red in B), and Cre (green in both A and B; yellow where both signals are approximately balanced). The Cre is found in the MNs, consistent with the reporter data. Arrows designate MNs with higher levels of Cre immunoreactivity, while arrowheads designate MNs with lower levels. Scale bars: 10 μm (A); 15 μm (B).

In initial pilot studies, AAV-Cre was injected into the facial nuclei of adult, wild-type mice. Cre was immunohistochemically detected in 1/2–2/3 of the MNs at 1-month post-injection. The expression was confined to the facial MNs and small, scattered subpopulations of other neurons along the needle tract. Almost all the facial MNs in or near sections with needle tracts expressed Cre, while progressively less MNs expressed Cre as the distance from the needle tract increased. This ability of AAV-Cre to very selectively infect neurons (e.g. Fig. 3) agrees with our experience using related AAV-2 vectors, and reports from other groups using AAV-2 constructs (e.g. Bartlett *et al.*, 1998; Kaspar *et al.*, 2002; Klein *et al.*, 2002).

To confirm that the Cre can excise floxed gene sequence in infected MNs, we used reporter mice in which active Cre leads to expression of beta-galactosidase (ROSA26 mice; Soriano, 1999) or GFP (Z/EG mice; Novak *et al.*, 2000). Both reporter signals were present in 1/2–2/3 of the facial MNs, and scattered neurons along the needle tract, by 2 weeks post-injection (Fig. 2B–D). The same fraction of MNs was also reporter-positive at 1 month post-injection (data not shown) suggesting that, as expected, essentially all MNs infected by the AAV-Cre injection are infected and expressing active levels of Cre by 2 weeks post-injection. As with the Cre immunohistochemistry, the percentage of MNs labeled in any given section was dependent on the section's proximity to the needle tract. No reporter signals were observed in: (i) non-neuronal cells [e.g. Supplementary material, Figs S1–S5 (note: both reporters can detect Cre activity in all non-neuronal cell types; e.g. supplementary Figs S5 and S6)]; (ii) wild-type mice identically injected with AAV-Cre; or (iii) uninjected reporter mice (data not shown).

### CNTFRα-dependent survival of adult facial MNs

There was no floxed vs. wild-type difference in facial MN number in uninjected mice (Fig. 4), consistent with the *lox* P sites not interfering with CNTFRα gene function. These naïve mice were all 7 months old when killed, while the injected mice described below ranged from 3 to 7 months old. Therefore, the floxed vs. wild-type differences in the injected mice do not reflect a differential age-related loss of MNs.

Facial nuclei of adult, floxed CNTFRα mice and age-matched (generally littermate), wild-type controls were injected with AAV-Cre or the PBS vehicle, and were perfused 1 week, 2 weeks or 1 month later. While no effect of AAV-Cre injection was observed at 1 or 2 weeks post-injection, at 1 month floxed mice contained significantly less facial MNs than the identically injected, wild-type controls (Fig. 4). In contrast, no floxed vs. wild-type difference was detected in vehicle-injected controls (Fig. 4). Therefore, AAV-Cre, which excises floxed genes in the MNs, leads to preferential loss of MNs containing floxed CNTFRα genes. At 1 month post-injection, AAV-Cre-injected wild-type mice contained less MNs than vehicle-injected wild-type mice (Fig. 4). Although not statistically significant at this time point, this effect reached significance by 4 months post-injection (Fig. 4). These results are consistent with previous reports of cellular toxicity associated with long-term exposure to Cre (e.g. Loonstra *et al.*, 2001; Kaspar *et al.*, 2002). As discussed below, they raise the interesting possibility that endogenous CNTF receptor signaling may promote the survival of MNs challenged by insult.

**Fig. 5:**
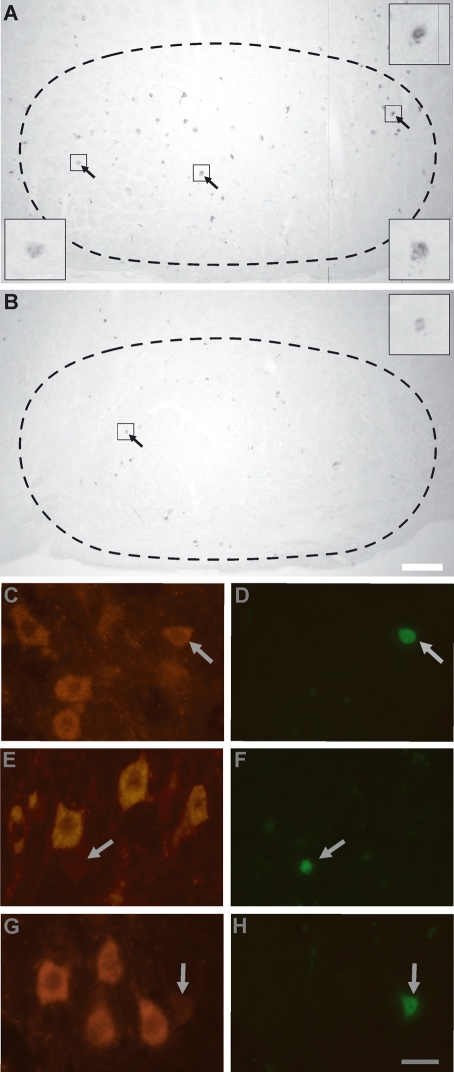
Cre and CNTFRα in facial MNs after AAV-Cre injection. Cre immunohistochemistry of wild-type (A) and floxed CNTFRα (B) mice perfused 1 month after AAV-Cre facial motor nucleus injection. Nuclear counterstaining (not shown) indicates that the label is primarily in the nuclei of the facial MNs (e.g. arrows), some of which are only partially in the plane of section. The examples illustrate the lower number of Cre-positive MNs remaining in floxed mice. Insets present arrowed nuclei at higher magnification. Broken lines delineate approximate facial motor nucleus borders. (C–H) While most Cre-expressing floxed MNs die by 4 months after AAV-Cre administration, almost all of the remaining few display no significant CNTFRα immunoreactivity (i.e. labeling equivalent to no primary antibody). CNTFRα immunohistochemistry (C, E, G) combined with Cre immunohistochemistry (D, F, H). Arrows designate Cre-positive wild-type (C and D) and floxed (E–H) MNs. Unlike the non-specific speckled Cre background, which does not correspond to cells, the larger and more intense Cre signals (arrows) correspond to MN nuclei. Scale bars: 100 μm (A and B); 25 μm (C–H).

Cre immunohistochemistry of wild-type mice perfused 1 week, 2 weeks or 1 month after AAV-Cre injection indicated that Cre expression rises in infected facial MNs during this period such that by 1 month post-injection about half of the MNs expressed detectable levels of Cre [366 ± 75 (mean ± SEM) at 1 week (*n* = 6); 470 ± 45 at 2 weeks (*n* = 6); 990 ± 75 at 1 month (*n* = 4)]. Comparison of these data to the reporter results (above) suggests that, while the reporters are more sensitive than the Cre immunohistochemistry at detecting the low-level Cre expression at 2 weeks post-injection, most, and possibly all, infected MNs eventually display both Cre immunohistochemical and reporter indicators by 1 month post-injection. At 1 and 2 weeks post-injection floxed mice displayed the same number of Cre-positive facial MNs as wild-type controls [351 ± 41 at 1 week (*n* = 6); 468 ± 72 at 2 weeks (*n* = 6)]. However, at the 1 month interval, when MN death is first detected (see above), the floxed mice displayed a 68% decrease in Cre-positive MNs relative to the controls [wild-type: 990 ± 75 (*n* = 4); floxed: 322 ± 102 (*n* = 4); *P* < 0.002; e.g. Fig. 5A and B]. The number of Cre-negative facial MNs in the same 1-month mice was calculated by subtracting the number of Cre-positive facial MNs from the number of total facial MNs obtained from counting CV-stained sections. In contrast to the number of Cre-positive MNs, the number of Cre-negative MNs was unaffected (wild-type: 618 ± 292; floxed: 734 ± 138), indicating that Cre-expressing MNs are selectively lost in the floxed mice.

**Fig. 6:**
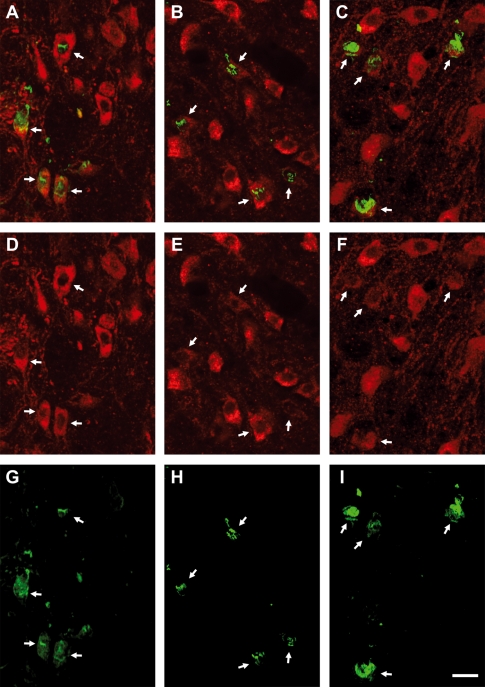
Examples of decreased CNTFRα immunoreactivity in Cre-expressing floxed facial MNs 2 weeks after AAV-Cre injection. Sections from wild-type (A, D, G) and floxed (B, E, H and C, F, I) mice immunohistochemically labeled for CNTFRα (red; in cytoplasm and processes of MNs) and Cre (green; in MN nuclei and, to a lesser extent, cytoplasm). Merged and individual signals from the same field are presented as columns with arrows designating examples of corresponding Cre-positive MNs. As quantitatively illustrated in Fig. 7, the Cre-expressing MNs in general display significantly reduced levels of CNTFRα immunoreactivity, but some still fall within the range seen with most wild-type MNs. The Cre signal includes an artifactual ‘smear’ that does not co-localize with other cells (based on counterstain) and, given its unidirectional nature in each field, presumably results from cell fragment displacement produced by the tissue sectioning. Scale bar, 20 μm.

**Fig. 7:**
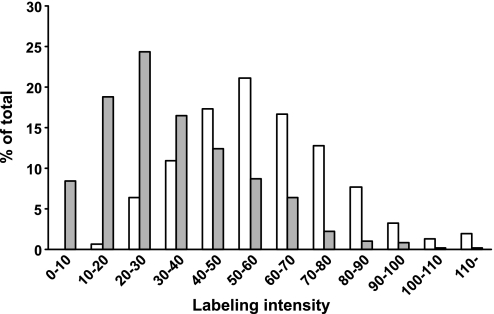
Decreased CNTFRα expression in Cre-expressing floxed CNTFRα MNs. MetaMorph software was used to analyze CNTFRα immunoreactivity levels in Cre-positive facial MNs of floxed (solid bars) and wild-type (open bars) mice killed 2 weeks after AAV-Cre injection. Cre-positive MNs were randomly selected while blind to genotype, and the average intensity of cytoplasmic CNTFRα labeling was determined for each cell. Background values (obtained from sampling adjacent tissue) were subtracted to produce a measure of CNTFRα expression (in arbitrary MetaMorph-generated units). The histogram presents the percentage of MNs in each labeling intensity category. It illustrates that the overall decrease in average CNTFRα immunoreactivity seen in the floxed mice (see text) results from decreased CNTFRα immunoreactivity in at least a large fraction of the cells. The wide range of labeling intensities observed in both floxed and wild-type mice may reflect real cell to cell differences in *in vivo* CNTFRα expression, but other factors, such as the different antibody concentrations encountered by MNs at different depths from the section surface, likely also contribute significantly.

We next quantified CNTFRα immunoreactivity in Cre-expressing MNs at 2 weeks post-injection in an effort to ‘catch’ some of the floxed neurons after their CNTFRα levels are detectably depleted but before the neurons die and are lost from the analysis. Examination of over 150 Cre-positive wild-type MNs (*n* = 4) and over 150 Cre-positive floxed MNs (*n* = 4) revealed a 43% decrease in average CNTFRα levels in the floxed MNs (*P* < 0.0001) that resulted from many of the floxed MNs expressing little if any significant CNTFRα and others still expressing CNTFRα levels in the range of the wild-type MNs at this time point (Figs 6 and 7), consistent with the asynchronous initiation of Cre expression in the MNs. In contrast, 4 months after AAV-Cre injection few Cre-positive floxed MNs were found and almost all of these were CNTFRα-negative (*n* = 2; e.g. Fig. 5C–H). Considered together, the above data indicate that the AAV-Cre-infected floxed MNs begin to lose CNTFRα by 2 weeks after injection, and many selectively die by 1 month, with few MNs surviving long-term without CNTFRα.

Finally, in order to examine the longer-term, CNTFRα-dependent survival of MNs in a context involving less Cre-related insult, we decreased the concentration of AAV-Cre by 90% and examined mice 4 months after AAV-Cre injection. As with the earlier experiments, floxed mice injected with ‘1/10 AAV-Cre’ contained significantly less MNs than identically injected wild-type controls (*P* < 0.001; Fig. 4). There was no significant effect of 1/10 AAV-Cre on wild-type MNs (*P* > 0.05), and the anova interaction term confirmed that 1/10 AAV-Cre led to significantly more MN loss in the floxed mice than in the wild-type controls (*P* < 0.01).

## Discussion

The present studies developed a floxed CNTFRα mouse line to conditionally disrupt the CNTFRα gene *in vivo*. In addition, an AAV-Cre vector was constructed and shown, with Cre immunohistochemistry and reporter mouse lines, to efficiently and selectively infect adult facial MNs *in vivo*, leading to MN Cre expression, excision of floxed gene sequence and decreased CNTFRα expression. AAV-Cre injection of floxed CNTFRα mice led to MN loss that was significantly greater than that in identically treated control mice, thereby revealing a role for endogenous CNTF receptor signaling in adult MN survival.

The use of AAV-Cre and floxed CNTFRα mice has several advantages. Most importantly, it avoids the perinatal death seen with unconditional CNTFRα knockout mice. Consequently, effects of CNTFRα gene disruption can be examined in the adult. By targeting facial MNs, that are not essential for the survival of the mice, MN loss can be followed over an extended period, while not confounded by mouse death. In contrast, the extent of MN loss observed in unconditional CNTFRα knockout mice (DeChiara *et al.*, 1995) may have been limited by the premature death of the mice. Therefore, it is not possible to meaningfully compare the extent of MN loss seen with the two models. It would be interesting to directly compare the adult and embryonic requirements for CNTF receptor signaling by selectively disrupting the CNTFRα gene in facial MNs as they are born embryonically. Unfortunately, to the best of our knowledge, this is not technically feasible at this time.

By initiating gene disruption in the adult, one can exclude potential development effects of gene disruption that are unrelated to ongoing adult functions. The present data indicate a role for CNTF receptor signaling in adult MN survival that is independent of any role it has in MN development or survival prior to adulthood. This promising finding is relevant to the design of any therapeutic interventions to treat adult MN diseases through manipulation of adult MN CNTF receptor signaling.

Consistent with previous reports showing neuron-selective infection by other AAV-2 vectors (e.g. Bartlett *et al.*, 1998; Kaspar *et al.*, 2002; Klein *et al.*, 2002), the present experiments with multi-label immunohistochemistry and those with two independent reporters indicate that AAV-Cre injection of the facial nucleus leads to floxed gene excision, which is highly restricted to neurons. Therefore, these data suggest that the preferential loss of MNs in CNTFRα floxed mice does not result from indirect effects of disrupting CNTF receptor signaling in non-neuronal cells. Instead, it appears most likely that CNTF receptor signaling by the MNs themselves plays a role in adult MN survival. This conclusion is further supported by the Cre immunohistochemistry results that indicate that most infected, floxed MNs die while uninfected floxed MNs are not affected, contrary to what would be expected if the critical CNTF receptor signaling occurred in non-neuronal cells. In other words, it is highly unlikely that any rare, undetected, non-neuronal cells with disrupted CNTF receptors would, with the vast majority of such non-neuronal cells still functioning normally, somehow lead to the death of most of the infected MNs but not affect the uninfected MNs. This realization that CNTF receptor signaling of adult MNs is likely critical to their own survival should be another important contribution to the design of therapeutic interventions targeting CNTF receptor signaling in the adult.

The CNTFRα-depleted MNs may die because CNTF receptors play an essential role in their survival even in the absence of insult, similar to what is observed in the embryo with the unconditional CNTFRα knockout (DeChiara *et al.*, 1995). However, reports that exogenous CNTF can protect MNs from traumatic and genetic insults (Sendtner *et al.*, 1990, 1992; Ikeda *et al.*, 1995; Sagot *et al.*, 1995; Pun *et al.*, 2006) raise the additional possibility that CNTF receptor signaling may serve as an endogenous neuroprotective mechanism. The present data are also consistent with this possibility. The trauma associated with the AAV-Cre injection procedure, while insufficient to kill a significant number of normal MNs [as indicated by the PBS-injected mice (Fig. 4)], may lead to death of MNs lacking neuroprotective CNTF receptors. Similarly, chronic exposure to high Cre concentrations can be toxic (Loonstra *et al.*, 2001; Kaspar *et al.*, 2002). Although the wild-type mice perfused either 1 month after AAV-Cre administration or 4 months after 1/10 AAV-Cre did not display a statistically significant decrease in MNs relative to vehicle-injected wild-type mice, the data, including the values from the wild-type mice 4 months after AAV-Cre injection, suggest that the long-term exposure to Cre can challenge MNs sufficiently to kill some. The greater loss of MNs in AAV-Cre-injected floxed mice relative to that in AAV-Cre-injected controls may result from an increased vulnerability of the floxed MNs due to the loss of CNTF receptors that would otherwise help protect the cells from toxic effects of Cre.

We targeted CNTFRα because CNTFRα disruption is the most comprehensive approach to determining the *in vivo* functions of endogenous CNTF receptor signaling. Thus, CNTFRα is essential for all known forms of CNTF receptor signaling, regardless of the participating ligands and signaling pathways (Davis *et al.*, 1993b; Elson *et al.*, 2000; Derouet *et al.*, 2004). Consequently, the present data do not address the individual ligand(s) or pathways involved in the CNTF receptor signaling that promotes adult MN survival. The CLC/CLF CNTF receptor ligand, which plays a substantial role in embryonic MN survival (Forger *et al.*, 2003), may play a similar role in adult MN survival. CNTF may also contribute, given that unconditional disruption of the CNTF gene leads to modest MN loss with aging (Masu *et al.*, 1993). Of course, multiple ligands may act together. Regardless, adult-onset disruption of individual ligands and pathways will be required to definitively address this issue.

Multiple endogenous growth factor systems function in a complex interaction to promote MN survival during development (Gould & Oppenheim, 2004). This is likely also the case in adulthood. In the present experiments most AAV-Cre-infected, floxed facial MNs died, such that no other endogenous growth factor mechanisms were able to save them. Therefore, it appears that, at least with the conditions and MN class involved here, adult endogenous CNTF receptor signaling plays an essential (i.e. non-redundant) role in the survival of most of the neurons.

*In vitro* work and unconditional knockout studies have identified many signaling proteins that are involved in MN survival. The approach characterized here should help reveal which of these candidates play an essential role in adult MN survival, *in vivo*. As discussed above, the selective genetic manipulation of adult facial MNs, which are not required for mouse survival, allows one to identify genes critical in adult MN survival independent of developmental effects, indirect effects of other cell types and premature death of the mice.

## Abbreviations

AAV: adeno-associated virus
CNTF: ciliary neurotrophic factor
CNTFRα: CNTF receptor α
Cre: Cre recombinase
CV: Cresyl violet
GFP: green fluorescent protein
LIF: leukemia inhibitory factor
LIFRβ: LIF receptor β
MN: motor neuron
PBS: phosphate-buffered saline
PFA: paraformaldehyde

## Supplementary material

The following supplementary material may be found on http://www.blackwell-synergy.com.

Fig S1 AAV-Cre-infected facial MNs in ROSA26^+/−^) reporter mice display discontinuous ‘specs’ of Xgal reporter staining in their processes, in addition to the much more intense soma labeling. Highmagnification photomicrograph of an AAV-Cre-infected facial MN (from experiment as in Fig. 2C).

Fig S2 Similar to Fig. S1.

Fig S3 Similar to Fig. S1.

Fig S4 Similar to Fig. S1.

Fig S5 Cre expression in non-neuronal facial nucleus cells of ROSA26^+/−^ reporter mice is clearly detected as distinctive Xgal staining.

Fig S6 Floxed gene excision detected in presumptive oligodendrocytes of ROSA26^+/−^ reporter mouse.
