# Supplementary material for: Conditional, genetic disruption of ciliary neurotrophic factor receptors reveals a role in adult motor neuron survival
Source: Eur J Neurosci. 2008 Jun;27(11):2830–7. doi: 10.1111/j.1460-9568.2008.06298.x (PMC2431126; doi:10.1111/j.1460-9568.2008.06298.x)
Supplement: Fig S1 — AAV-Cre-infected facial MNs in ROSA26+/−) reporter mice display discontinuous ‘specs’ of Xgal reporter staining in their processes, in addition to the much more intense soma labeling. Highmagnification photomicrograph of an AAV-Cre-infected facial MN (from experiment as in Fig. 2C). [file ejn0027-2830-SD1.doc]

**Fig. S1**. AAV-Cre-infected facial motor neurons in ROSA26^+/-^ reporter mice display discontinuous “specs” of Xgal reporter staining in their processes, in addition to the much more intense soma labeling. High magnification photomicrograph of an AAV-Cre infected facial MN (from experiment as in Figure 2C). The section has also been CV stained. The infected MN (dark blue in center-top of figure) contrasts with a non-infected MN (purple CV stain only in upper-right of figure). Examples of MN processes (with weak CV stain) are indicated by arrowheads. The Xgal labeling of the processes is indicated by open arrows. CV-stained, non-neuronal cells (which are much smaller than the motor neurons) do not display Xgal staining (examples designated by solid arrows). Note: Xgal staining of such cells (in other experiments where Cre is induced in such cells [see Supplementary Fig. S5]) presents as a distinctive blue signal both in and surrounding the cells. Scale bar = 10 µm.
