# Supplementary material for: Conditional, genetic disruption of ciliary neurotrophic factor receptors reveals a role in adult motor neuron survival
Source: Eur J Neurosci. 2008 Jun;27(11):2830–7. doi: 10.1111/j.1460-9568.2008.06298.x (PMC2431126; doi:10.1111/j.1460-9568.2008.06298.x)
Supplement: Fig S2 — Similar to Fig. S1. [file ejn0027-2830-SD2.doc]

**Fig. S2**. AAV-Cre-infected facial motor neurons in ROSA26^+/-^ reporter mice display discontinuous “specs” of Xgal reporter staining in their processes, in addition to the much more intense soma labeling. High magnification photomicrograph of an AAV-Cre infected facial MN (from experiment as in Figure 2C). The section has also been CV stained. Examples of MN processes (with weak CV stain) are indicated by arrowheads. The Xgal labeling of the processes is indicated by open arrows. The other specs of Xgal staining presumably correspond to processes of this MN or other infected MNs which, unlike the more proximal dendrites, predictably do not stain as much with CV. CV-stained, non-neuronal cells (which are much smaller than the motor neurons) do not display Xgal staining (examples designated by solid arrows). Note: Xgal staining of such cells (in other experiments where Cre is induced in such cells [see Supplementary Fig. S5]) presents as a distinctive blue signal both in and surrounding the cells. Scale bar = 10 µm.
