# Supplementary material for: Conditional, genetic disruption of ciliary neurotrophic factor receptors reveals a role in adult motor neuron survival
Source: Eur J Neurosci. 2008 Jun;27(11):2830–7. doi: 10.1111/j.1460-9568.2008.06298.x (PMC2431126; doi:10.1111/j.1460-9568.2008.06298.x)
Supplement: Fig S4 — Similar to Fig. S1. [file ejn0027-2830-SD4.doc]

**Fig. S4**. AAV-Cre-infected facial motor neurons in ROSA26^+/-^ reporter mice display discontinuous “specs” of Xgal reporter staining in their processes, in addition to the much more intense soma labeling. More examples as presented in Supplemental figures 1-3. Scale bars = 10 µm.
