# Supplementary material for: Conditional, genetic disruption of ciliary neurotrophic factor receptors reveals a role in adult motor neuron survival
Source: Eur J Neurosci. 2008 Jun;27(11):2830–7. doi: 10.1111/j.1460-9568.2008.06298.x (PMC2431126; doi:10.1111/j.1460-9568.2008.06298.x)
Supplement: Fig S5 — Cre expression in non-neuronal facial nucleus cells of ROSA26+/− reporter mice is clearly detected as distinctive Xgal staining. [file ejn0027-2830-SD5.doc]

**Fig. S5**. Cre expression in non-neuronal facial nucleus cells of ROSA26^+/-^ reporter mice is clearly detected as distinctive Xgal staining. CV and Xgal staining of facial nucleus tissue from a ROSA26^+/-^ reporter mouse which carries a GFAP-Cre gene construct driving Cre expression in GFAP-expressing cells. Arrows designate examples of affected cells (presumptive astrocytes). Note: ROSA26^+/-^ reporter mice detect Cre-induced gene excision in all cell types including oligodendrocytes (e.g., Supplementary Fig. S6), microglia and macrophages. Scale bar = 10 µm.
