# Supplementary material for: Conditional, genetic disruption of ciliary neurotrophic factor receptors reveals a role in adult motor neuron survival
Source: Eur J Neurosci. 2008 Jun;27(11):2830–7. doi: 10.1111/j.1460-9568.2008.06298.x (PMC2431126; doi:10.1111/j.1460-9568.2008.06298.x)
Supplement: Fig S6 — Floxed gene excision detected in presumptive oligodendrocytes of ROSA26+/− reporter mouse. [file ejn0027-2830-SD6.doc]

**Fig. S6**. Floxed gene excision detected in presumptive oligodendrocytes of ROSA26^+/-^ reporter mouse. Xgal staining of corpus callosum of ROSA26^+/-^ reporter mouse with floxed gene excision in presumptive oligodendrocytes, which frequently are aligned in characteristic rows (designated by arrows). Scale bar = 25 µm.
